# Comparative Genomic and Phylogenetic Analysis of Chloroplasts in *Citrus paradisi* Mac.cv. Cocktail

**DOI:** 10.3390/genes16050544

**Published:** 2025-04-30

**Authors:** Qun Wu, Yun Zhu, Shipei Zheng, Jiajun Wang, Huilin Cheng, Haimin Chen, Weidong Zhu

**Affiliations:** 1 Quzhou Academy of Agriculture and Forestry Sciences, Quzhou 324003, China; wuq_2006@aliyun.com (Q.W.); wangjjqznky@sina.com (J.W.); chenghl@126.com (H.C.); 2 Key Laboratory of Plant Secondary Metabolism and Regulation of Zhejiang Province, College of Life Sciences and Medicine, Zhejiang Sci-Tech University, Hangzhou 310018, China; zhuyunzstu@outlook.com (Y.Z.); 13437985756@163.com (S.Z.)

**Keywords:** chloroplast genome, *Citrus paradisi* Mac. cv. Cocktail, phylogenetic analysis

## Abstract

**Background:** *Citrus paradisi* Mac. cv. Cocktail is globally valued for its abundant nutrients and bioactive compounds, particularly in tropical and subtropical regions. A novel albino phenotype mutant of *C. paradisi* Mac. cv. Cocktail (designated WT) was identified in Quzhou and subsequently named *C. paradisi* Mac. cv. Cocktail mosaic mutant (MT). To distinguish *C. paradisi* Mac. cv. Cocktail from conventional grapefruit cultivars and to elucidate genomic differences between WT and MT, this study conducted a comprehensive comparison of their chloroplast genomes with those of previously reported *Citrus* species. **Methods:** The complete chloroplast genomes of WT and MT were assembled through Illumina PE150 sequencing, enabling detailed comparative genomic and evolutionary studies. **Results:** The results revealed that both WT and MT chloroplast genomes exhibit a conserved quadripartite structure. Each genome measures 160,186 base pairs in length, with a uniform GC content of 38.5%. Annotation revealed 138 genes (91 protein-coding, 10 rRNA, 37 tRNA), 82 repeats, and A/U-biased codons. Genome boundaries and genome comparison showed WT and MT were identical but divergent from other *Citrus*. The 52 conserved protein-coding genes showed comparable selection pressures in both WT and MT. Phylogenetically, WT and MT are closely related and are distinguished from all of the traditional grapefruits. **Conclusions:** The albino phenotype of MT is unrelated to chloroplast variations. Chloroplast genomics supports *C. paradisi* cv. Cocktail’s differentiation from conventional grapefruits. This study expands the chloroplast genomic resources for *Citrus* and establishes a theoretical framework for future research on *C. paradisi* cv. Cocktail and related varieties.

## 1. Introduction

*Citrus paradisi* Mac. cv. Cocktail is a delightful hybridization of Citrus maxima Burm and Citrus reticulata Blanco [1] which is widely cultivated and distributed in Zhejiang Province, China. *C. paradisi* Mac. cv. Cocktail exhibits a high degree of sweetness and is rich in a variety of nutrients beneficial to human health, making it suitable for the production of fermented fruit juices [2]. Additionally, grapefruit peel is a source of diverse secondary metabolites, predominantly flavonoids and phenols. Studies have demonstrated that these compounds can regulate human metabolic processes and exhibit a range of biological activities, including antioxidant and anti-cancer properties [2,3]. Consequently, grapefruit peel has the potential to develop natural pharmaceuticals.

Chloroplasts are ubiquitous in the plant kingdom and participate in key biochemical processes, including photosynthesis [4]. They constitute a vital organelle within plant cells. The chloroplast genome is characterized by its typical double-stranded circular DNA molecule, which is frequently utilized for species identification owing to its relatively slow evolutionary rate [5]. The chloroplast genome features a closed, double-stranded DNA structure, comprising a large single-copy (LSC) region, a small single-copy (SSC) region, and two inverted repeat (IR) regions. This configuration ensures a stable, conserved tetrad structure [6,7]. Consequently, the distinctive characteristics of the chloroplast genome render it a suitable DNA barcode for conducting phylogenetic and biodiversity analyses of species [8,9,10]. Chloroplast genome sequences have facilitated the classification of numerous closely related species and the analysis of mutated species [11,12,13,14,15]. Plastome analysis such as chloroplasts is also widely used at the population level, such as Mendelian pea pan-plastome analysis, which provides important resources for pea breeding and variety improvement [16]. For plants of significant value, such as Pinus gerardiana [17], Acer ukurunduense [18] and Lemna turionifera [19], cpDNA sequencing also provides a genetic basis for resource conservation and artificial cultivation.

In this study, we generated and characterized the complete chloroplast genomes of both the wild-type *C. paradisi* Mac. cv. Cocktail (WT) and its albino mosaic mutant counterpart (MT). Our comparative analysis was motivated by two fundamental hypotheses: (1) the chloroplast genome of *C. paradisi* Mac. cv. Cocktail exhibits distinct characteristics that differentiate it from other grapefruit varieties. (2) Structural variations in the chloroplast genomes of WT and MT variants may underpin their observed phenotypic divergence. To investigate these hypotheses, we performed comprehensive bioinformatic analyses encompassing codon usage pattern analysis, simple sequence repeat (SSR) locus identification, inverted repeat (IR) boundary characterization, whole genome comparison genomics and phylogenetic reconstruction. The primary objective of this investigation is to elucidate the systematic positioning of *C. paradisi* Mac. cv. Cocktail within the Citrus genus phylogenetic framework. Additionally, we seek to determine whether candidate genes associated with the phenotypic differentiation between wild-type and mutant phenotypes are localized to the chloroplast genome. This research contributes to the expansion of the chloroplast genomic resources for citrus species while providing critical insights into the molecular mechanisms potentially governing albinism development in horticulturally significant citrus cultivars.

## 2. Materials and Methods

### 2.1. Plant Materials and DNA Extraction

**Figure 1:**
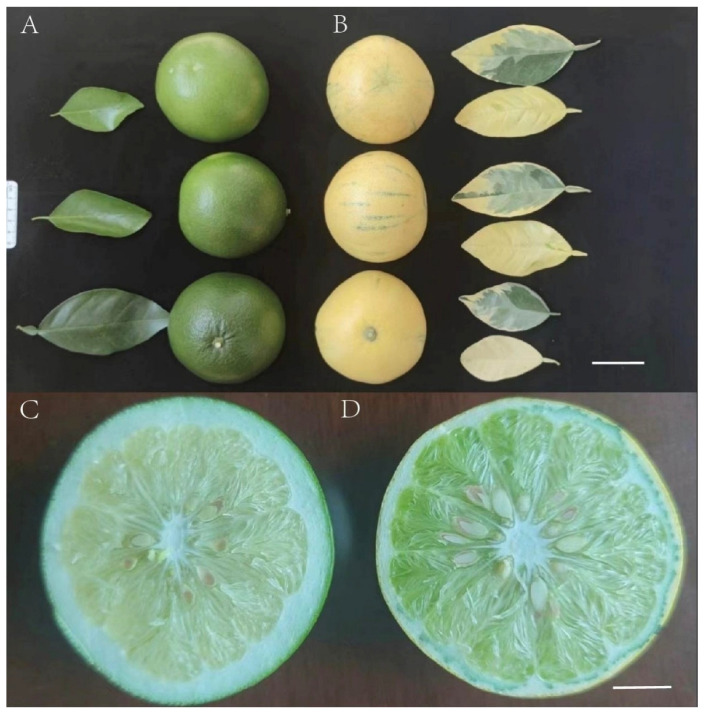
Characteristics of *C. paradisi* Mac. cv. Cocktail (WT) and *C. paradisi* Mac. cv. Cocktail mosaic mutant (MT): (**A**) (WT) and (**B**) (MT) represent the external morphological diagrams of the fruits and leaves of WT and MT, respectively (bar = 5 cm), and (**C**) (WT) and (**D**) (MT) represent the sectional views of their fruits, respectively (bar = 2 cm).

Leaf samples from the wild-type (WT) and mutant-type (MT) were obtained from the Quzhou Academy of Agricultural and Forestry Sciences, Zhejiang, China (29.073231° N, 119.043128° E). The phenotypic differences were significant (Figure 1). Genomic DNA was extracted from the leaves using the cetyltrimethylammonium bromide (CTAB) method. The integrity and purity of the DNA were assessed using agarose gel electrophoresis and a NanoDrop spectrophotometer (Thermo Fisher Scientific, Wilmington, DE, USA), while a Qubit 3.0 fluorometer (Thermo Fisher Scientific, Carlsbad, CA, USA) was employed to precisely measure the DNA concentration.

### 2.2. Assembly and Annotation of Chloroplast Gene Sequences

After constructing the library, sequencing was performed on the Illumina platform PE150. DNA sequencing was performed using Illumina adapter primers: P5 (5′-AATGATACGGCGACCACCGAGATCTACAC-3′) and P7 (5′-CAAGCAGAAGACGGC ATACGAGAT-3′). The original sequencing data were first filtered and trimmed using the Fastp program, and then input into Getorganelle [20] for assembly using the *Citrus sunki* chloroplast genome as the reference sequence (GenBank accession no. MT767776.1). Subsequently, the assembled genome was annotated using Geneious Prime 2024.0.5 [21] and manually corrected using Geneious Prime 2024.0.5 to verify the accuracy and completeness of the annotation. Methods for verifying annotation results encompass gene structure visualization and genome variation analysis. Finally, the GeSeq website (https://chlorobox.mpimp-golm.mpg.de/geseq.html, accessed on 17 April 2025) [22] was utilized to generate the circular maps of each chloroplast genome. After generating the chloroplast genome map, we utilized ODGRAW to obtain the map, then imported the map into Adobe Illustrator 2021 software for adjustments, and finally exported it in JPG format. The chloroplast genome sequence was deposited in GenBank (accession numbers: PP_940535).

### 2.3. Analysis of Simple Sequence Repeats (SSR)

The chloroplast simple sequence repeats (SSRs) are a special class of mononucleotide tandem repeats, which are characterized by high abundance and polymorphism. The variability in the type, number, and location of these repeats between species renders them valuable as molecular markers [23]. For the analysis of SSRs, the Misa website (https://webblast.ipk-gatersleben.de/misa/, accessed on 17 April 2025) [24] was employed. After uploading the sequence file, the minimum repeat numbers of mononucleotides, dinucleotides, trinucleotides, tetranucleotides, pentanucleotides, and hexanucleotides were manually set to 10, 6, 5, 4, 3, and 3, respectively.

### 2.4. Analysis of Codon Usage Bias

Codons encoding the same amino acid are designated as synonymous codons, which exhibit different usage frequencies. This phenomenon is referred to as codon usage bias. In the absence of codon usage bias, the relative synonymous codon usage (RSCU) value of a codon is equal to 1. An RSCU value greater than 1 for a codon suggests a higher usage frequency. Given that this metric can partially reflect codon bias, it is frequently utilized in research and analysis pertaining to codon bias [25]. To analyze the codon usage bias in WT and MT, the Codon W application [26] should be employed, and the FASTA files should be input to calculate RSCU values separately. The data should be organized and the findings should be presented in an Excel spreadsheet.

### 2.5. Comparative Analysis

The mVISTA website (http://genome.lbl.gov/vista/mvista/submit.shtml, accessed on 17 April 2025) [27] was utilized to assess the divergence in the chloroplast genomes among the WT and MT, as well as four additional *Citrus* species (*Citrus x paradisi, Citrus maxima, Citrus natsudaidai*, and *Citrus x tangelo*). *Citrus x paradisi* served as the reference sequence, with the WT sequence positioned second and the MT sequence third. Subsequently, the sequences of the related species were uploaded in sequential order. The analysis results were distributed via email links, and the PDF files were subsequently downloaded and modified using Adobe Illustrator 2021 software. Additionally, CPJSdraw (http://cloud.genepioneer.com:9929/#/tool/alltool/detail/335, accessed on 17 April 2025) [28] was employed for the IR analysis and visualization of the junctions and marginal regions within the six grapefruit chloroplast genomes.

### 2.6. Analysis of Selective Pressures in the Evolution

CPGANA-toolkit software was utilized to compare differences in chloroplast genome sequences. First, the common CDS region of all chloroplast genome sequences was extracted, and *Triphasia trifolia* (LC_794908.1), which is in the same family as *C. paradisi* Mac. cv. Cocktail, was selected as the reference species, and its GenBank file was downloaded. The common CDS extracted from other species were aligned with the reference species. Pairwise alignments of sequences were conducted at the codon level, and .axt files were automatically generated. Then, the calculation results from KaKs Calculator were extracted, and the heat map was created after filtering and imputing NA values. The software automatically generated PNG images.

### 2.7. Phylogenetic Analysis

To ascertain the phylogenetic relationships of WT and MT, we downloaded 10 chloroplast sequences of *Citrus* genus and 1 species of Sapindaceae from the NCBI GenBank database, with *Atalantia buxifolia* (GenBank accession no. OK_356595.1) as an outgroup. Using MEGA 11 [29], the phylogenetic relationships of 13 plant chloroplast genomes were analyzed by maximum likelihood (ML) method, and 1000 bootstrap repeats were performed. Firstly, we used the Mafft alignment function of Genius software to align all sequences, and then used MEGA 11 to draw a phylogenetic tree.

## 3. Results

### 3.1. Characterization of Chloroplast Genomes

**Figure 2:**
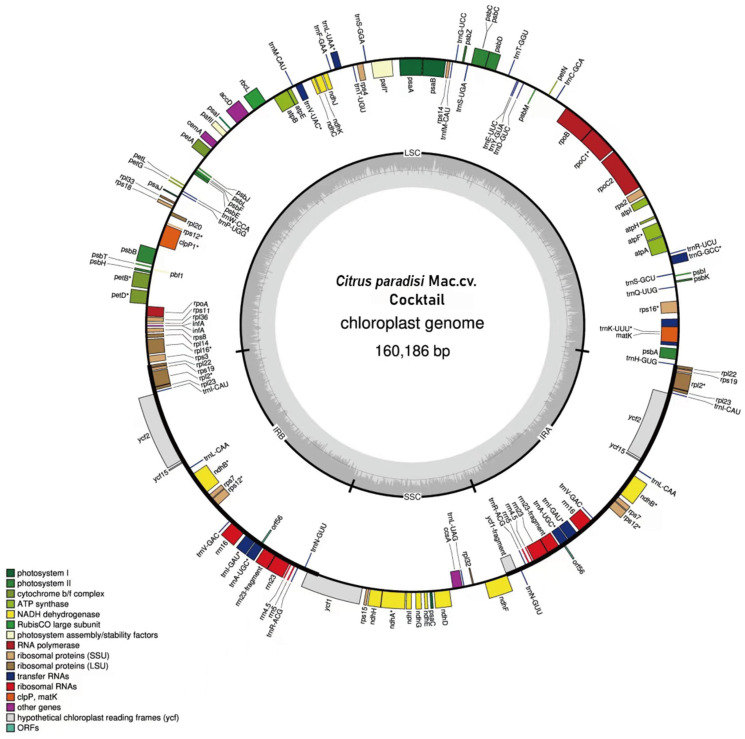
Gene map of the chloroplast genome from *C. paradisi* Mac. cv. Cocktail. Genes depicted within the circumference are transcribed in a clockwise direction, while those situated outside are transcribed counterclockwise. Dark gray in the inner circle corresponds to GC content. LSC, large single-copy region; SSC, small single-copy region; IRA and IRB, two inverted repeat sequences. Label intron-containing genes with *.

The chloroplast genomes of WT and MT exhibited no differences in sequence length, GC content, and gene composition. The chloroplast genome possessed structural characteristics typical of most angiosperms [30], comprising a large single-copy region, a small single-copy region, and two inverted repeat sequences (IRa and IRb) (Figure 2). The GC content across these four regions was 36.8%, 33.3%, 42.9% and 42.9%, respectively, suggesting a high degree of conservation in the two inverted repeat sequences. The nucleotide composition of the chloroplast genome was A (31.1%), T (30.5%), C (19.0%), and G (19.5%), with a total GC content of 38.5%. The total length of the chloroplast genome sequence was 160,168 bp, with the large single-copy (LSC) region measuring 87,791 bp, the small single-copy (SSC) region measuring 18,395 bp, and the inverted repeats (IRa and IRb) region measuring 27,000 bp.

A total of 138 genes were annotated in the gene map, comprising 91 protein-coding genes, 10 rRNA, and 37 tRNA. The protein-coding genes included 12 genes encoding the small subunit of the ribosome, namely *rps12, rps7, rps19, rps3, rps8, rps11, rps18, rps14, rps4, rps2, rps16*, and *rps15*. Additionally, there were 10 genes encoding the large subunit of the ribosome: *rpl23*, *rpl2*, *rpl22*, *rpl16*, *rpl14*, *rpl36*, *rpl20*, *rpl33*, and *rpl32*. Five genes (*psaA*, *psaB*, *psaC*, *psaI*, and *psaJ*) associated with photosystem I were identified. A total of 13 genes pertained to photosystem II: *psbT*, *psbH*, *psbE*, *psbF*, *psbC*, *psbD*, *psbL*, *psbI*, *psbJ*, *psbM*, *psbZ*, *psbK*, and *psbA*. Furthermore, six genes were involved in ATP synthesis within the electron transport chain: *atpA*, *atpB*, *atpE*, *atpF*, *atpH*, and *atpI*.

### 3.2. Analysis of Simple Sequence Repeats (SSRs)

SSRs are prevalent in plant chloroplast genomes and are used to classify plants, identify mutations, and elucidate phylogenetic connections [31]. The analytical results demonstrated identical SSR loci (type, number, and distribution) between WT and MT chloroplast genomes. A total of 82 repeats were identified, comprising 78 single-base repeats, 1 three-base repeat, 2 four-base repeats and 1 five-base repeat, with no two-base repeats present (Table 1). Chloroplast SSR markers from *C. paradisi* Mac. cv. Cocktail constitute a valuable resource for the identification of *Citrus* and offer a robust tool for assessing and analyzing grapefruit germplasm in future studies [32].

### 3.3. IRs Boundary Analysis

**Figure 3:**
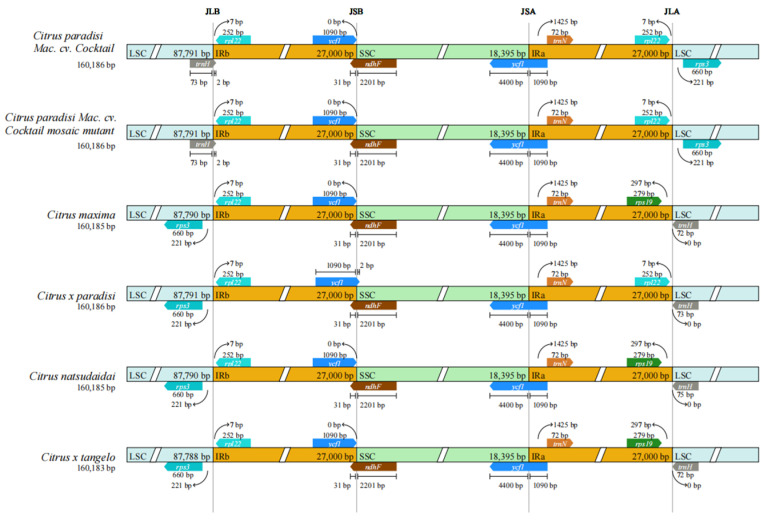
Comparison between LSC, SSC, and IRs junction boundaries in the chloroplast genomes of six *Citrus* species: *Citrus natsudaidai*, *Citrus maxima*, *Citrus x tangelo*, *Citrus x paradisi*, *C. paradisi* Mac. cv. Cocktail and *C. paradisi* Mac. cv. Cocktail mosaic mutant.

The chloroplast genome structure is delineated by four distinct vertical lines, JLB, JSB, JSA, and JLA, which represent the boundaries of different regions (Figure 3). The figure revealed that the gene lengths and locations are approximately consistent across the six species. Within the chloroplast genome boundaries of these six *Citrus* plants, the WT and MT, as well as *Citrus x tangelo* and *Citrus maxima*, exhibited identical characteristics. This indicates that there is no difference in the IRs boundary analysis between WT and MT. However, a notable distinction exists in the IRs boundary when comparing these two types with the chloroplasts of the other four citrus species. The *trnH* and *rps3* genes in the six plants were located in the LSC region, whereas the *ycf1* gene was positioned at the SSC/IRa and SSC/IRb boundaries. However, a complete *rps19* gene was present in the IRa region of *Citrus natsudaidai*, *Citrus maxima*, and *Citrus x tangelo*, whereas *Citrus x paradisi*, *C. paradisi* Mac. cv. Cocktail, and *C. paradisi* Mac. cv. Cocktail mosaic mutant lacked this gene.

### 3.4. Codon Usage Analysis

The left and right columns of Table 2 show the codon usage analysis data for WT and MT, respectively (Table 2). The codon usage patterns were largely similar with minor variations. Both WT (left) and MT (right) possess a total of 53,395 codons. Leucine (Leu) codons are the most frequently utilized, whereas tryptophan (Trp) codons are the least. Among all amino acid-encoding codons, AAA (WT) and UUU (MT) are the most abundant, occurring 2274 and 2276 times, respectively, with RSCU values of 1.3256 and 1.2116. CGC (WT) and GCG (MT) codons are the least frequently utilized, appearing only 298 and 310 times, with RSCU values of 0.4813 and 0.7368, respectively. Furthermore, methionine (Met) and tryptophan (Trp) each have a single corresponding codon, ATG and TGG, respectively, with RSCU values of one, indicating no bias. Notably, 32 out of the 64 codons in their chloroplast genome exhibited preference codons with frequencies exceeding one.

### 3.5. Genome Comparison

**Figure 4:**
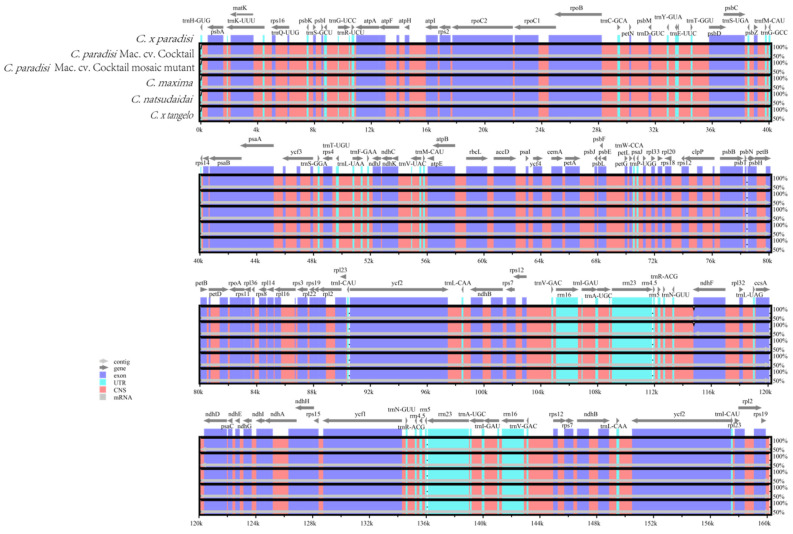
Six chloroplast genomes were aligned globally using *Citrus x paradisi* as a reference. Each coordinate represents a region of the chloroplast genome. The vertical axis represents the percentage of the aligned region sequence similarity.

The mVISTA software was used for comparative analysis of the chloroplast genomes between WT and MT and four related species. The reference sequence employed was that of the *Citrus x paradisi* chloroplast genome. Collectively, the variation among the six chloroplast genome sequences was not substantial (Figure 4). The chloroplast genome sequences of WT and MT were completely identical. Within the 115–117 kb region, discrepancies were observed between WT and MT and the four related species, but the difference was not significant. Comparative analysis of the chloroplast genome sequences of *C. paradisi* Mac. cv. Cocktail and related species revealed genetic differences. These findings may provide important insights for taxonomic and evolutionary research of these species [33].

### 3.6. Selective Pressures in the Evolution

Synonymous and non-synonymous substitutions of protein-coding genes are common during gene evolution. A codon undergoing a synonymous substitution retains the same amino acid, whereas a non-synonymous substitution results in a different amino acid. The rates of synonymous (Ka) and non-synonymous (Ks) substitutions, along with their ratios (Ka/Ks), are frequently utilized to assess the evolutionary rate between gene sequences, thereby discerning the presence of selective pressure during evolution. Typically, equivalence of Ka and Ks suggests that the gene is neutral with respect to selective pressure. When Ka exceeds Ks, the gene is inferred to be under positive selection pressure. When Ka is less than Ks, the gene is considered to be under purifying (negative) selection pressure. If Ks equals zero during calculation, rendering the Ka/Ks ratio undefined, the value for this scenario is conventionally set to zero.

**Figure 5:**
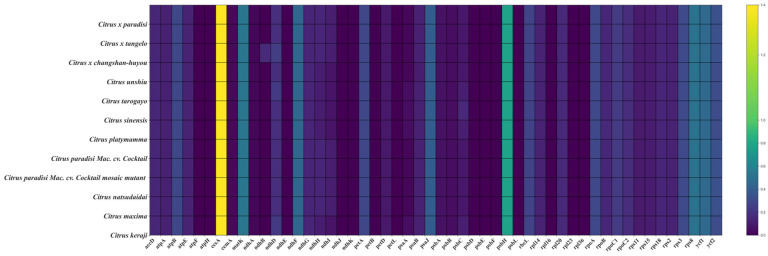
Selective pressure analysis of 52 common protein-coding genes in 12 chloroplast genomes.

A total of 52 common protein-coding genes were extracted from the chloroplast genomes of WT, MT, and ten other *Citrus* genera for analysis of selective pressure. The analysis revealed that the majority of the 12 chloroplast genomes exhibited Ka/Ks ratios below 1, indicating that the majority of gene mutations were likely deleterious, and only a subset of genes exhibited Ka/Ks ratios higher than one during purification, including *ccsA* and *psbH* (Figure 5). Furthermore, no difference in selection pressure was detected between WT and MT across the 52 conserved protein-coding genes.

### 3.7. Phylogenetic Analysis

**Figure 6:**
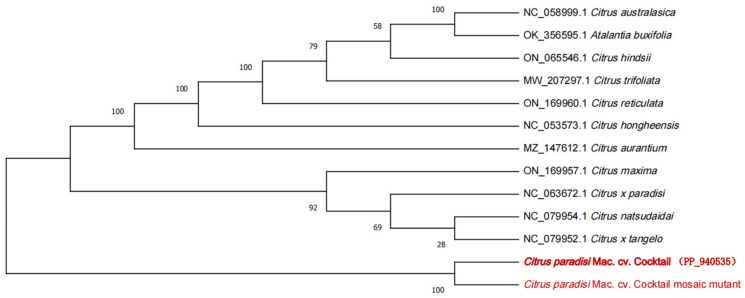
The ML phylogenetic tree showing the relationship between *C. paradisi* Mac. cv. Cocktail and *C. paradisi* Mac. cv. Cocktail mosaic mutant and other species of *Citrus* based on the complete plastid genomes catenated dataset. Numbers in each the node indicated the bootstrap support values.

Eleven Rutaceae species were selected for constructing the phylogenetic tree with WT and MT, including 10 species related to the *Citrus* genus and one outgroup species from the *Atalantia* genus of Rutaceae. The phylogenetic tree delineated the taxonomic relationships between *C. paradisi* Mac. cv. Cocktail and other species. The result indicated that WT and MT are closely related and are distinguished from all of the traditional grapefruits (Figure 6).

## 4. Discussion

In this study, the chloroplast genomes of WT and MT were sequenced, assembled, and annotated. Concurrently, comparative analysis was conducted with 11 previously reported species. The chloroplast of *C. paradisi* Mac. cv. Cocktail exhibited a structure analogous to that of most angiosperms, featuring a quadripartite 160,168 bp arrangement comprising LSC, SSC, IRa, and IRb [34]. The GC content of four regions was 36.8%, 33.3%, 42.9%, and 42.9%, respectively. The GC content in the IR regions was higher than that in the LSC and SSC regions, suggesting that the two inverted repeats were highly conserved. A total of 138 functional genes and non-coding RNA were found in the *C. paradisi* Mac. cv. Cocktail chloroplast, including 91 protein-coding genes, 10 rRNA, and 37 tRNA. Among them, 25 genes contained 1 intron (15 protein-coding genes and 8 tRNA genes), and 2 genes contained 2 introns (*pafI* and *clpP1*).

SSRs are extensively utilized as molecular markers in genetic map construction, genetic diversity research, and genetic relationship analysis [35,36]. A total of 164 SSR sequences were identified in the chloroplast genomes of WT and MT, with no observed differences between them. Notably, single-base A/T SSRs are the most prevalent, constituting up to 93.9% of the total, with the highest number of repetitions. The examination of codon usage patterns is of great significance for deciphering evolutionary pressures and advancing genetic research [37]. In this study, 64 sites with RSCU values greater than one and 60 sites with RSCU values less than one were identified. Among the codons exhibiting RSCU values above one, 53.13% terminate in base A/U, while 46.87% terminate in base C/G. Codon usage in both WT and MT demonstrated a preference for termination in A/U.

In the analysis of IRs boundary variation, the IRs boundaries of WT and MT were indistinguishable, but markedly distinct from those of the other four species. The *trnH* genes in WT and MT were located at the LSC/IRb boundary, whereas those in the other four species were located at the LSC/IRa boundary. Concurrently, within the IRa region, similar to *Citrus x paradisi*, the *rps19* gene was absent. A comparable phenomenon was observed in comparative genomic analyses. WT and MT exhibited a high degree of similarity, but showed subtle variation with the other four species in the 115–117 kb region. Genetic distance analysis further revealed that *C. paradisi* Mac. cv. Cocktail and its variants were closely related, then differentiated *C. paradisi* Mac. cv. Cocktail from traditional grapefruits.

In selection pressure analysis, ω > one is typically interpreted as indicative of a positive selection effect, implying that certain beneficial mutations are currently subject to selection. When ω = one, it indicates neutrality and implies a state of neutral evolution. If 0 < ω < 1, it is considered to be indicative of purifying or negative selection. The Ka/Ks ratios of *C. paradisi* Mac. cv. Cocktail and its closely related species were less than one, indicating strong purification selection pressure and conservation of amino acid sequences [38,39].

The chloroplast genomes of WT and MT exhibited no disparities regarding sequence length, GC content, gene composition, simple repetitive sequences, IR boundaries, codon usage bias, and comparative genomic analysis. Based on the chloroplast genome analysis, no differences were observed between WT and MT at the chloroplast genomic level. The results do not support our original hypothesis that the albino phenotype of MT was not a consequence of variations within the chloroplast genome. However, albinism may stem from epigenetic DNA methylation or mutations in genes associated with chloroplast function, which requires further study [40,41]. Furthermore, analyses of the IR boundaries, comparative genomics, and phylogenetics revealed that WT and MT were distinct from other *Citrus* species. The results conclusively validate our hypothesis that chloroplast genome analysis could be used to distinguish *C. paradisi* Mac. cv. Cocktail from other grapefruit varieties.

## 5. Conclusions

Comprehensive chloroplast genome sequencing, assembly, and annotation of wild-type *C. paradisi* Mac. cv. Cocktail (WT) and its albino mosaic mutant (MT) revealed conserved quadripartite architecture (LSC/SSC/IRa/IRb boundaries) with identical 160,186 bp length, 38.5% GC content, and 138 functional genes (91 protein-coding, 10 rRNA, 37 tRNA). Comparative analysis with published *Citrus* species demonstrated strict sequence conservation (100% identity) between WT and MT, absence of structural variations, and identical repetitive element profiles (82 mononucleotide repeats dominating, with 78 SNPs and minor contributions from tri-/tetra-/pentanucleotide repeats). Codon usage analysis confirmed A/T-ending codon bias consistent with *Citrus* chloroplast genomes. Notably, the absence of chloroplast genome variation between phenotypically divergent WT and MT suggests that the albinism phenotype likely arises from nuclear gene mutations or epigenetic modifications (e.g., DNA methylation) affecting chloroplast development. Systematic comparisons through inverted repeat boundary analysis, phylogenetic reconstruction, and genome-wide homology assessments further established that *C. paradisi* Mac. cv. Cocktail possesses distinct chloroplast genomic features enabling clear taxonomic discrimination within the *Citrus* genus. These findings enrich citrus chloroplast genomic resources while providing critical insights into cytoplasmic inheritance patterns and molecular mechanisms underlying horticultural traits. This work establishes a foundation for future studies on chloroplast genome influenced phenotypic variation in *Citrus*, offering practical applications for cultivar identification, evolutionary studies, and functional investigations into plastid-related albinism etiology.

## Figures and Tables

**Table 1:** Comprehensive analysis of the distribution and diversity of simple sequence repeats (SSRs) within the chloroplast genomes of the wild-type (WT) and mosaic mutant (MT) varieties of *C. paradisi* Mac. cv. Cocktail.

SSR Type (Number of Copies)	SSR Sequence	Number of Copies																			Total Number
		03	04	05	06	07	08	09	10	11	12	13	14	15	16	17	18	19	20	21	
Mononucleotide (78)	A/T	-	-	-	-	-	-	-	18	16	14	10	6	6	-	3	3	-	-	1	77
	C/G	-	-	-	-	-	-	-	1	-	-	-	-	-	-	-	-	-	-	-	1
Trinucleotide (1)	AAT/ATT	-	-	-	1	-	-	-	-	-	-	-	-	-	-	-	-	-	-	-	1
Tetranucleotide (2)	AAAT/ATTT	-	2	-	-	-	-	-	-	-	-	-	-	-	-	-	-	-	-	-	2
Pentanucleotide (1)	AAAAT/ATTTT	1	-	-	-	-	-	-	-	-	-	-	-	-	-	-	-	-	-	-	1

**Table 2:** The relative synonymous codon usage (RSCU) values of *C. paradisi* Mac. cv. Cocktail (WT) and *C. paradisi* Mac. cv. Cocktail mosaic mutant (MT).

Amino Acid	Codon	Number	RSCU	Amino Acid	Codon	Number	RSCU
**Ter**	UAA	1111	1.1929	**Ter**	UAA	1158	1.1634
	UAG	750	0.8053		UAG	839	0.8429
	UGA	933	1.0018		UGA	989	0.9936
**Ala**	GCA	490	1.0925	**Ala**	GCA	470	1.1171
	GCC	430	0.9588		GCC	386	0.9174
	GCG	318	0.7090		GCG	310	0.7368
	GCU	556	1.2397		GCU	517	1.2288
**Cys**	UGC	456	0.8057	**Cys**	UGC	486	0.8541
	UGU	676	1.1943		UGU	652	1.1459
**Asp**	GAC	409	0.5550	**Asp**	GAC	450	0.5810
	GAU	1065	1.4450		GAU	1099	1.4190
**Glu**	GAA	1432	1.3978	**Glu**	GAA	1429	1.3707
	GAG	617	0.6022		GAG	656	0.6293
**Phe**	UUC	1435	0.7990	**Phe**	UUC	1481	0.7884
	UUU	2157	1.2010		UUU	2276	1.2116
**Gly**	GGA	899	1.3565	**Gly**	GGA	910	1.4577
	GGC	401	0.6051		GGC	363	0.5815
	GGG	714	1.0773		GGG	625	1.0012
	GGU	637	0.9611		GGU	599	0.9596
**His**	CAC	410	0.6274	**His**	CAC	425	0.6574
	CAU	897	1.3726		CAU	868	1.3426
**Ile**	AUA	1441	0.9763	**Ile**	AUA	1347	0.9596
	AUC	1139	0.7717		AUC	1053	0.7502
	AUU	1848	1.2520		AUU	1811	1.2902
**Lys**	AAA	2274	1.3256	**Lys**	AAA	2200	1.3652
	AAG	1157	0.6744		AAG	1023	0.6348
**Leu**	CUA	737	0.8600	**Leu**	CUA	802	0.9086
	CUC	627	0.7316		CUC	687	0.7783
	CUG	520	0.6068		CUG	463	0.5245
	CUU	1051	1.2264		CUU	1099	1.2451
	UUA	1103	1.2870		UUA	1143	1.2949
	UUG	1104	1.2882		UUG	1102	1.2485
**Met**	AUG	893	1.0000	**Met**	AUG	807	1.0000
**Asn**	AAC	801	0.6185	**Asn**	AAC	824	0.6317
	AAU	1789	1.3815		AAU	1785	1.3683
**Pro**	CCA	739	1.1934	**Pro**	CCA	777	1.1702
	CCC	654	1.0561		CCC	696	1.0482
	CCG	432	0.6976		CCG	487	0.7334
	CCU	652	1.0529		CCU	696	1.0482
**Gln**	CAA	1163	1.4046	**Gln**	CAA	1048	1.4067
	CAG	493	0.5954		CAG	442	0.5933
**Arg**	AGA	1175	1.8977	**Arg**	AGA	1114	1.8723
	AGG	676	1.0918		AGG	606	1.0185
	CGA	698	1.1273		CGA	655	1.1008
	CGC	298	0.4813		CGC	318	0.5345
	CGG	466	0.7526		CGG	463	0.7782
	CGU	402	0.6493		CGU	414	0.6958
**Ser**	AGC	532	0.6829	**Ser**	AGC	520	0.6266
	AGU	695	0.8922		AGU	657	0.7917
	UCA	880	1.1297		UCA	1022	1.2316
	UCC	810	1.0398		UCC	931	1.1219
	UCG	694	0.8909		UCG	689	0.8303
	UCU	1063	1.3646		UCU	1160	1.3979
**Thr**	ACA	704	1.1887	**Thr**	ACA	657	1.1521
	ACC	554	0.9354		ACC	600	1.0522
	ACG	422	0.7125		ACG	380	0.6664
	ACU	689	1.1634		ACU	644	1.1293
**Val**	GUA	716	1.2079	**Val**	GUA	705	1.1634
	GUC	467	0.7879		GUC	484	0.7987
	GUG	409	0.6900		GUG	441	0.7277
	GUU	779	1.3142		GUU	794	1.3102
**Trp**	UGG	757	1.0000	**Trp**	UGG	754	1.0000
**Tyr**	UAC	702	0.6689	**Tyr**	UAC	711	0.6749
	UAU	1397	1.3311		UAU	1396	1.3251

## Data Availability

The plastome data of the *C. paradisi* Mac. cv. Cocktail have been submitted to GenBank (accession: PP_940535).
